# Initial evidence of abnormal brain plasticity in anorexia nervosa: an ultra-high field study

**DOI:** 10.1038/s41598-022-06113-x

**Published:** 2022-02-16

**Authors:** Edoardo Pappaianni, Bianca Borsarini, Gaelle E. Doucet, Ayelet Hochman, Sophia Frangou, Nadia Micali

**Affiliations:** 1 Department of Psychiatry, Faculty of Medicine, University of Geneva, 2 Rue Verte, 1205 Geneva, Switzerland; 2 Boys Town National Research Hospital, Omaha, NE USA; 3 Department of Psychology, St. John’s University, Queens, NY USA; 4 Department of Psychiatry, Icahn School of Medicine at Mount Sinai, New York, NY USA; 5 Department of Psychiatry, University of British Columbia, Vancouver, BC Canada; 6 Great Ormond Street Institute of Child Health, University College London, London, UK; 7 Department of Pediatrics, Gynecology and Obstetrics, Faculty of Medicine, University of Geneva, Geneva, Switzerland

**Keywords:** Psychiatric disorders, Magnetic resonance imaging

## Abstract

Anorexia Nervosa has been associated with white matter abnormalities implicating subcortical abnormal myelination. Extending these findings to intracortical myelin has been challenging but ultra-high field neuroimaging offers new methodological opportunities. To test the integrity of intracortical myelin in AN we used 7 T neuroimaging to acquire T1-weighted images optimized for intracortical myelin from seven females with AN (age range: 18–33) and 11 healthy females (age range: 23–32). Intracortical T_1_ values (inverse index of myelin concentration) were extracted from 148 cortical regions at ten depth-levels across the cortical ribbon. Across all cortical regions, these levels were averaged to generate estimates of total intracortical myelin concentration and were clustered using principal component analyses into two clusters; the outer cluster comprised T_1_ values across depth-levels ranging from the CSF boundary to the middle of the cortical regions and the inner cluster comprised T_1_ values across depth-levels ranging from the middle of the cortical regions to the gray/white matter boundary. Individuals with AN exhibited higher T_1_ values (i.e., decreased intracortical myelin concentration) in all three metrics. It remains to be established if these abnormalities result from undernutrition or specific lipid nutritional imbalances, or are trait markers; and whether they may contribute to neurobiological deficits seen in AN.

## Introduction

In the central nervous system, myelin is a cholesterol rich extension of oligodendrocytes that coats axons, i.e. the long processes of neurons^1,2^. Axon myelination in the nervous system enables fast, saltatory electrical impulse propagation through action potentials modulation. Myelin can be thought of as an insulator, which boosts the conduction speed of the action potential along the axonal body, increasing the neural signal transmissibility from neuron to neuron, ultimately sustaining adequate cerebral intra-and-inter regional communication^2^. Motor, sensory, and cognitive functions of the nervous system all require rapid impulse propagation relying on an adequate axonal myelination^2,3^. Not only does myelin surround white matter bundles, it also plays a key role in cortical gray matter. Intracortical myelin is most prevalent in the deeper layers of the cortex, and it seems to enhance cortical function by fine-tuning timing and synchrony of neural networks^4^. Being particularly sensitive to experience^3^ and in continuous development throughout life^5^, intracortical myelin participates in brain plasticity and remodeling. This is evidenced by the fact that myelin content is associated with cognitive function, and that higher levels of intracortical myelin are associated with better performance on cognitive tasks^5^. Therefore, it is safe to assume that an abnormal concentration of intracortical myelin may play a role in psychiatric disorders. This is the case with schizophrenia, where a loss of intracortical myelin has been shown^3,6,7^.

Animal studies have provided consistent support for an adverse impact of undernutrition on myelination, given the potentially detrimental effects of long-lasting starvation on white matter (WM) composition^8,9^. Despite the lack of equivalent investigations in humans, myelin seems to be sensitive to micro-and-macro nutrient deficiencies: in fact, evidence in the literature suggests that iron is directly involved in myelin production as a required co-factor for cholesterol and lipid biosynthesis^10^. Authors have suggested that inflammation, oxidative stress, and loss of oligodendrocytes might all play a role^11–13^.

Anorexia nervosa (AN) is a severe psychiatric disorder with one of the highest long-term mortality rates of all psychiatric disorders^14–16^. It affects predominantly women, emerging typically during adolescence and early adulthood^17^. The core features of the disease are an intense fear of gaining weight, a body-image distortion leading to weight loss, behaviors and strategies aimed at weight loss and resulting in a state of severe malnutrition^18^. There is growing evidence of the impact of AN on the brain, both on structure and function^19–25^. In particular, WM microstructure alterations, gray and WM volume abnormalities have been shown in patients with AN in both acute and chronic phases^20–22,26–29^.

Given that animal models suggest a myelin decrease following malnutrition, the evidence of intracortical myelin abnormalities in other psychiatric disorders, and its maturational pattern (that extends into late adolescence and adulthood)^30–32^, it is reasonable to hypothesize a myelin deficit in patients with AN. However, since in AN WM investigations have been almost-exclusively carried out with standard Magnetic Resonance Imaging (MRI) acquisition sequences, a direct quantitative detection of myelin concentration in-vivo has been challenging^33^.

New techniques based on ultra-high field MRI imaging^34–38^ may address this gap in the literature, allowing visualization of the brain at submillimeter resolution^39^ and examination of myelin in a more fine-grained fashion. In view of the fact that the human cortex is approximately 2–4 mm thick, standard spatial resolution (1 mm) does not allow for proper detection of intracortical myelin^40^. Moreover, evidence shows that depth-dependent intracortical myelin properties obtained by ultra-high field MRI are reliable and consistent with ex-vivo data^34,39,40^. Based on this premise, in our preliminary study we aimed to measure intracortical myelin in patients with AN and controls, capitalizing on a newly validated method able to ascertain depth-dependent intracortical myelin organization with an ultra-high field 7 T scanner^39,40^. Specifically, using an ad-hoc acquisition sequence and data analysis procedure, in-vivo T_1_ values (in milliseconds) as inverse measure of intracortical myelin^39^ were extracted and compared in a sample of women with AN and healthy controls. To our knowledge, this type of investigation of cortical myelo-architecture has never been carried out in AN. Moreover, the use of quantitative MRI to measure actual tissue parameters (such as T_1_ values) prevents the risk of tissue or hardware-related bias that may impact generalizability of results^41^. We hypothesized that individuals with AN would show higher T_1_ values (i.e., lower intracortical myelin concentration) compared to controls. We also explored whether the differences in intracortical myelin concentration would be particularly visible in the outermost cortical vs. innermost depth-levels in AN. We further investigated any relationships between disorder-related variables and intracortical myelin.

## Results

### Socio-demographic and illness-related data

Individuals with AN did not differ from controls in terms of age. As expected, individuals with AN had a significantly lower Body Mass Index (BMI) compared to controls (Welch t = 6.057, p < 0.001) (Table 1). Osmolality levels were within the normal range for 4/7 (57.1%) individuals with AN, while three patients had slightly higher values than the normal range^42^. Duration of illness in individuals with AN ranged between 1 and 21 years. No current psychiatric comorbidity, or past psychiatric diagnosis was identified. Only one participant reported psychotropic medication use (venlafaxine).

**Table 1:** Descriptive statistics:

	AN patients	Controls	Group differences
Age (years)	25.14 (5.21)	27.18 (3.28)	ns^+^
BMI (kg/m^2^)	17.34 (0.63)	23.03 (3.02)	t = 6.057, p < 0.001
Osmolality (mmol/kg)*	289.71 (16.53)		
EDE: global score	2.54 (0.93)		
EDE: restraint	2.49 (1.34)		
EDE: eating concern	1.89 (1.42)		
EDE: shape concern	3.20 (0.99)		
EDE: weight concern	2.57 (1.03)		

*Osmolality index expressed in milliosmoles/kg.

Mean (standard deviation) and group differences for age and BMI are shown. Average osmolality index, EDE's global score, restraint, eating concern, shape concern and weight concern subscales score are reported for individuals with AN only. EDE, Eating Disorder Examination.

^+^p > 0.05.

**Table 2:** Global T_1_ values across individuals with AN and controls: Analysis of Covariance.

	AN	Controls	Group differences^a^
T_1_ values (ms)—total intracortical myelin concentration	1858.96 (28.08)	1809.95 (43.47)	F(1,15) = 6.01, p = 0.03, η^2^ = 0.29

Mean (standard deviation) of average T_1_ values across all ten cortical depth-levels. Group differences in terms of F value (degrees of freedom), p value and effect size are shown.

^a^Analysis corrected for age.

### Global T1 values

**Figure 1:**
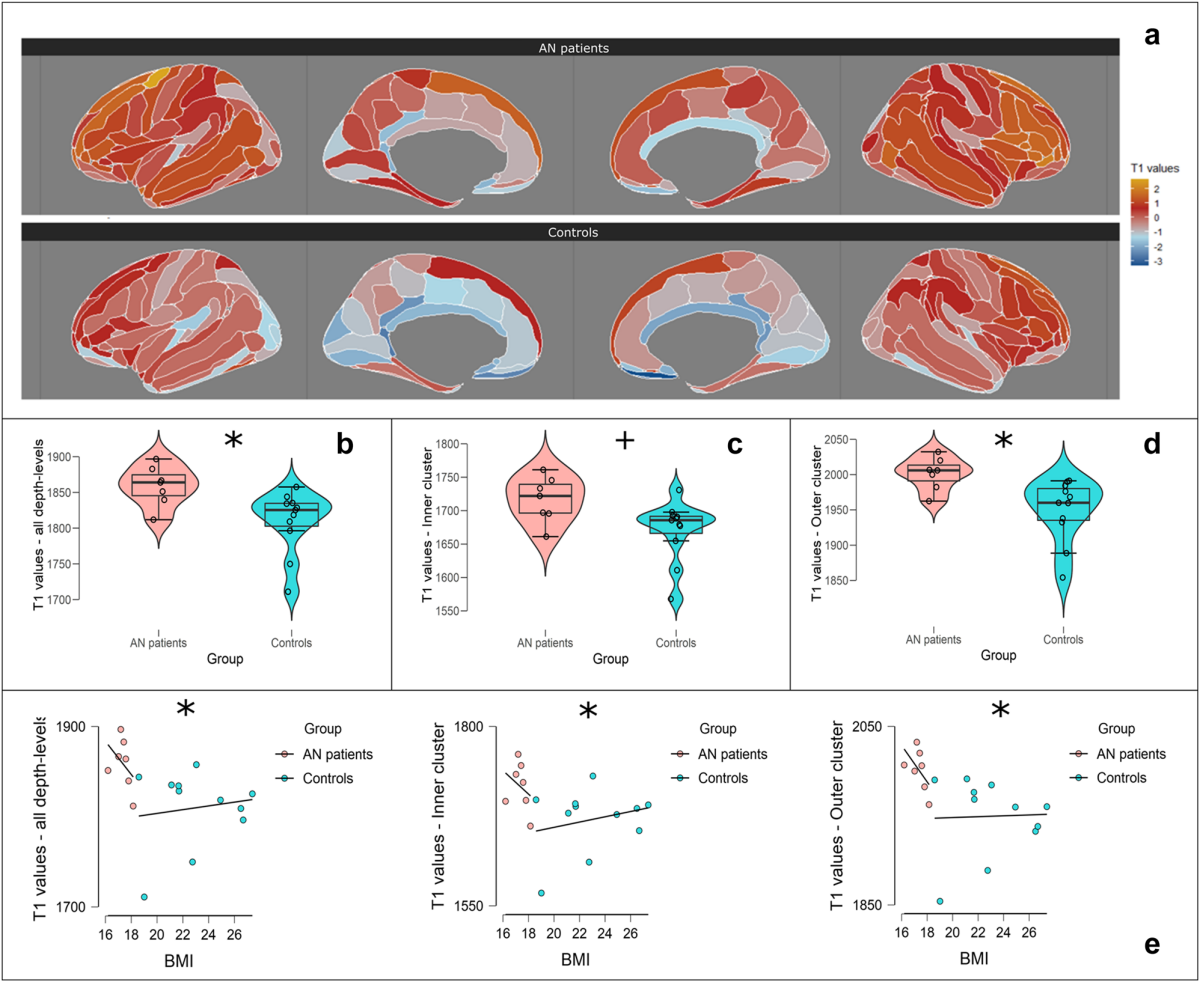
**(a)** Standardized total T_1_ values map for AN patients (top) and controls (bottom). Maps are based on the Destrieux’s Atlas and show T_1_ values’ average across 10 depth-levels per each Region-of-Interest (ROI) in AN patients and healthy controls. For illustrative purposes, the distribution of T1 values was rescaled to a distribution with mean = 0. (**b)** Boxplot with violin and jitter elements displaying distribution of T_1_ values (in ms) as intracortical myelin concentration index averaged across all 10 cortical depth-levels for AN patients and controls; (**c)** boxplot with violin and jitter elements displaying distribution of inner cluster’s T_1_ values (in ms) for AN patients and controls; (**d)** boxplot with violin and jitter elements displaying distribution of outer cluster’s T_1_ values (in ms) for AN patients and controls; (**e)** scatterplots between BMI and T_1_ values (in ms) in all depth-levels, inner and outer clusters in AN patients and controls. For illustrative purposes groups are shown separately, although the statistical significance symbol refers to the analysis on overall population. T_1_ values distributions between AN patients and controls and relationship between T1 values and BMI. *p < 0.05; ^+^p = 0.05.

Participants with AN and controls had equal variances distribution for total T_1_ values (in milliseconds) (Levene’s Test p > 0.05). A statistically significant difference emerged between groups for T_1_ values (F(1,15) = 6.95, p = 0.03, η^2^ = 0.29). In detail, patients with AN showed higher T_1_ values than controls (Mean Difference MD = 48.55 ms, Standard Error SE = 19.80, 95% Confidence Intervals (C.I.) = 6.34, 90.77, Cohen’s d = 1.26). These differences also emerged in the bootstrap post-hoc comparison (MD = 48.28 ms, Bias = −0.406, SE = 17.29, 95% bias corrected accelerated C.I. = 15.28, 85.14). See Table 2 and Fig. 1b for all details. A graphical representation of total T_1_ values maps is available in Fig. 1a. Images were rendered with the *ggsegDesterieux* (https://github.com/LCBC-UiO/ggsegDesterieux) and *ggseg*^43^ (https://lcbc-uio.github.io/ggseg/) packages via RStudio v. 1.2.50. working on R v.4.0.4.

Based on the structural indications provided by the Principal Component Analysis (PCA), we averaged the T_1_ values from depth levels 1 to 5 independent of ROIs to form a composite measure of intracortical concentration in an “inner cluster” (i.e., cluster extending from the middle of the cortical ribbon to the gray matter/white matter boundary). The corresponding measures from depth levels 6 to 10 were also averaged independent of ROIs to generate a composite measure of intracortical concentration in an “outer cluster” (i.e., cluster extending from the middle of the cortical ribbon to the gray matter/CSF boundary). The mean cortical concentration of the inner and outer clusters were included in two separate set of analyses. See Table 3 and Fig. 1c,d for details about inner and outer cluster distributions.

**Table 3:** T_1_ values in the inner and outer cluster across individuals with AN and controls: Analysis of Covariance.

	AN	Controls	Group differences^a^
T_1_ values (ms)—inner cluster	1716.54 (34.20)	1670.53 (45.04)	F(1,15) = 4.32, p = 0.05, η^2^ = 0.22
T_1_ values (ms)—outer cluster	2001.39 (23.23)	1949.38 (43.69)	F(1,15) = 7.61, p = 0.01, η^2^ = 0.34

Mean (standard deviation) of average T_1_ values across cortical depth-levels 1–5 (inner cluster) and 6–10 (outer cluster). Group differences in terms of F value (degrees of freedom), p value and effect size are shown.

^a^Analyses corrected for age.

### Inner cluster

Patients with AN and controls had an equal variance distribution in T_1_ values within the inner cluster (Levene’s Test p > 0.05). The ANCOVA reported a difference approaching statistical significance between groups (F(1,15) = 4.32, p = 0.05, η^2^ = 0.22). In the inner cluster, AN patients showed higher T_1_ values in respect to controls (MD = 44.07 ms, SE = 21.20, 95% C.I. −1.11, 89.26, Cohen’s d = 1.07). This result was confirmed by a bootstrapped post-hoc comparison based on 1000 replications (MD = 43.33 ms, Bias = −2.08, SE = 19.26, 95% bias corrected accelerated C.I. = 8.93, 86.58).

### Outer cluster

Both groups reported equal variance distribution in T_1_ values within the outer cluster (Levene’s Test p > 0.05). The ANCOVA showed a significant effect of group on T_1_ values (F(1,15) = 7.61, p = 0.01, η^2^ = 0.34). In outer cluster, individuals with AN showed significantly higher T_1_ values than controls (MD = 53.03 ms, SE = 19.29, 95% CI = 12.05, 95.01, Cohen’s d = 1.42), confirmed by a bootstrapped post-hoc test (MD = 51.29, Bias = −1.46, SE = 15.93, 95% bias corrected accelerated C.I. = 26.66, 91.62).

### Correlations between illness-related variables and intracortical myelin

At the general level BMI was negatively correlated with global T_1_ values (rho = −0.68, p = 0.003, C.I. = −0.87, −0.31), T_1_ values in the inner (rho = −0.57, p = 0.017, C.I. = −0.82, −0.14) and outer cluster (rho = −0.75, p < 0.001, C.I. = −0.90, −0.44). Visually, such correlation seems more prominent in AN patients, although within group analyses did not reveal any significant correlations (p > 0.05) (Fig. 1e for scatter-plots). In addition, we explored the relationship between intracortical myelin concentration, osmolality and duration of illness in AN individuals. Osmolality marginally correlated with total myelin concentration (rho = 0.79, p = 0.05, C.I. = 0.08, 0.97) and with myelin in the inner cluster (rho = 0.89, p = 0.01, C.I. 0.43, 0.98), while it was less correlated to myelin concentration in outer cluster (rho = 0.50, p = 0.27, C.I. −0.41, 0.91). Duration of illness did not correlate with total intracortical myelin concentration, inner and outer cluster myelin concentration (all p > 0.05).

## Discussion

In this preliminary study, we used an innovative method based on 7 T ultra-high field MRI to examine depth-dependent intracortical myelin in a population of women with AN. In accordance with our hypothesis, women with AN showed higher T_1_ values across the cortical ribbon. These differences were stronger in the five outermost intracortical depth-levels, whilst they were less evident in the five inner intracortical layers. Such results may index an intracortical myelin deficit in women with AN compared to healthy controls.

The use of a high-resolution MRI scanner allowed for the first time to use T_1_ values as a proxy to investigate depth-dependent intracortical myelin organization in-vivo. Our results are consistent with previous findings of reduced myelin concentration in AN^27,44^, and go beyond these, showing that such a deficit is widespread and mainly affects the outer depth-levels dependent intracortical myelin organization. Travis and colleagues^27^ reported a relationship between decreased fractional anisotropy (FA, defined as a marker of WM integrity) and decreased relaxation time R1 (considered as an index of myelin content) in AN adolescents. This suggests that observed WM differences in AN might be due to reductions in myelination^10,45^. However, as stated recently by Barona and colleagues in their meta-analysis^46^, differences in WM could be due to several factors, including a larger axon diameter, lower packing density of fibers, altered membrane permeability as well as a myelin deficit^47^. In fact, Travis and colleagues^27^ also underlined that the most significant WM disruptions occurred where the axons showed the largest diameter and a thicker myelin shell (i.e. the corticospinal tract and the corpus callosum)^21^.

Whereas in our study higher T_1_ values were found in patients with AN, in a recent study^44^ performed with a 3 T MRI scanner an opposite pattern emerged, i.e., patients with AN showed lower T_1_ relaxation times (i.e. time constant indicating how long the protons need to return to the equilibrium state^41^) than controls in both gray matter and WM. Nonetheless, the authors suggested that these differences could be related to starvation-driven myelin loss^44^. Although the two interpretations seem counterintuitive, it is worth highlighting that T_1_ relaxation time is strictly dependent on the physical properties of the underlying tissue^41^, including free water content^48,49^ and concentration and types of macromolecules^50^ such as iron content^49^ and myelin^51^. Thus, to date, such methodologies based on in-vivo quantitative MRI do not allow confirmation that between group differences may be due to various factors other than myelin content. Nevertheless, it is known that while water content is directly proportional to T_1_ time, iron content and myelination are inversely related to T_1_^41^. This is confirmed by the fact that T_1_ relaxation time is generally longer in WM, which is myelin dense^41^. While Boto and colleagues^44^ did not exclude the impact of water content in the measurement of relaxation times, in our study hydration level was not a confounder. Moreover, the relationship between T_1_ and myelin has also been confirmed by ex-vivo data^34,39,40,51^, even in populations characterized by strong myelin loss such as multiple sclerosis^52,53^.

It is known that myelin assembling persists during development and in adult life^54–57^. Animal studies highlight that the process of myelin lipid remodeling is continuous, and that the rate of change of such lipids is different throughout life^58,59^. Myelin membranes are known to have a very high lipid-to-protein ratio, with lipids accounting for at least 70% of the dry weight^2^. Myelin construction therefore requires a large amount of lipids, including cholesterol, galactolipids, plasmalogen, and fatty acids^60^. In consideration of the reduced caloric intake, decrement in intracortical myelination in AN may mirror oligodendrocytes’ dysfunctions due to lack of micronutrients necessary for the synthesis of specific lipids. Nevertheless, further studies targeted on AN are needed to confirm such a claim.

A myelin deficit may also play a fundamental role in the symptomatic manifestations of AN. Animals studies suggest intracortical myelin is related to cortical functions and behavior^61^, and changes in myelin preceded changes in behavior^54,55^. Therefore several authors have suggested that intracortical myelin might be a marker of plasticity in the cortex^40,61^. Myelin damage, demyelination or oligodendrocyte loss results in a global malfunction in many neurological conditions such as multiple sclerosis, leukodvstrophies, Alzheimer’s disease, stroke, cerebral palsy, traumatic brain-spinal injury and cognitive decline^62,63^. Therefore intracortical myelin loss might contribute to the neurocognitive deficits of patients with AN such as cognitive flexibility, weak central coherence, emotional processing difficulties^64^.

Further investigations will need to study closely the relationship between neurocognitive differences and myelin concentration in AN.

### Strengths and limitations

This preliminary study is the first to investigate depth-dependent intracortical myelin organization in individuals with AN. Using the high spatial resolution afforded by 7 T imaging in conjunction with a previously validated method for intracortical myelin estimation^40^ we obtained information about T_1_ values used as a proxy for intracortical myelin concentration across the cortical ribbon. Given the small sample size and the lack of specific a-priori assumptions, we chose not to focus on specific ROIs and this limitation should be addressed in future studies. The small sample size represents the main limitation but the current findings albeit preliminary encourage further studies in larger and more fully characterized samples. Osmolality as an index of hydration was collected in AN patients only, precluding us from including it as a covariate in our main analysis. However, correlation between osmolality and myelin concentration in the outermost depth-levels was weaker than within the inner cluster in AN patients, and only two out of seven patients with AN had abnormal osmolality levels indicative of dehydration, hence it is unlikely that dehydration fully confounds our findings. At the time of the study, the patients were all in the acute phase of illness, but duration of illness varied across our sample. Although no relationship emerged between illness duration and variables of interest, there might have been heterogeneities in the clinical sample. Although we are aware of the aforementioned limitations, we believe that our study provides promising evidence of altered intracortical myelin organization in AN.

## Conclusions

In this preliminary study, we leverage a novel method for the in-vivo examination of T_1_ values as an inverse index of intracortical myelin organization in AN. We found generally higher T_1_ values in women with AN compared to healthy women, mirroring a widespread lower myelin concentration. These differences might be due to malnutrition and starvation, and general brain atrophy, or might be trait markers of the illness. However, the specific mechanisms (nutritional or structural) that might lead to myelin loss, and most importantly if this loss is reversible, need to be elucidated further. Moreover given the newly hypothesized role of metabolic factors in the etiology of AN^65^, whether a premorbid abnormality in myelin structure exists needs specific investigation. In a more speculative fashion, our study also raises the possibility that cognitive, visuo-spatial (and other) difficulties seen in active AN might be the result of altered intracortical myelin. Despite limitations, our findings provide an important foundation on which to build future studies. If confirmed, these results could shed new light on the neural basis of AN and on brain effects of the illness.

## Methods

### Participants

The study was carried out at the Icahn School of Medicine at Mount Sinai, New York. Participants were recruited amongst patients from the Mount Sinai Eating and Weight Disorders Program, the Mount Sinai Psychiatry service and from the community via flyers and adverts. Initial screen for eligibility was conducted over the phone by a research coordinator. We included female participants, with an age range from 18 to 45, who were able to provide informed consent and who spoke and understood English fluently. Patients had to have: (a) a diagnostic statistical manual of mental disorders, 5th edn (DSM-5)^66^ diagnosis of AN; (b) a body mass index (BMI) between 15.5 and 18.5; (c) a history of food restriction of more than 1 year; (d) to be medically stable. Healthy controls were screened for eligibility and had to have no history of mental health disorders (including eating disorders) and no chronic medical conditions.

Exclusion criteria for all participants included any contraindication to the MRI examination (i.e., embedded or implanted metallic bodies and claustrophobia) and pregnancy.

Participants who met initial criteria for the study were invited for an in-person assessment. After providing informed consent, participants completed initial study measures and completed a urine pregnancy test. Seven female adults with current AN and 11 healthy female adults were included.

The study was approved by the institutional review committee of the Icahn School of Medicine at Mount Sinai (ISMMS). All research was performed in accordance with relevant guidelines and regulations. Patients and controls provided informed consent to participate.

### Measures

Socio-demographic data were collected using a short questionnaire. Weight and height were measured objectively to calculate BMI (kg/m^2^) (Table 1). Data were collected on socioeconomic status, duration of illness, and current medications in individuals with AN.

The Eating Disorder Examination interview version16.0D (EDE^67^) was used for diagnostic purposes. The EDE is a semi-structured interview considered the “gold standard” for assessing eating disorder symptoms. The EDE assesses a range of eating disorder features and yields a global score of eating disorder symptoms (Table 1).

The SCID Screening Module^68^ was administered to screen participants for other psychiatric disorders conditions.

AN patients underwent a blood draw in order to establish their level of osmolality (i.e. an hydration index based on molecular weight/1 L water presence ratio expressed in milliosmoles per kilogram).

### MRI acquisition

Patients and controls were scanned at the ISMMS using a 7 T MR scanner (Magnetom, Siemens Healthcare) with a 32-channel with a Nova head coil (Nova Medical, Wilmington MA). An ultra-high resolution MP2RAGE sequence was used to acquire whole brain T1-weighted images. Acquisition parameters consisted of 0.5 mm isotropic resolution, repetition time (TR) = 5000 ms, echo time (TE) = 5.75 ms, inversion time (TI) TI1/TI2 = 900 ms/2780 ms, 224 axial slices with slab thickness 11.5 cm, field-of-view 224.5 × 203 × 112 mm^3^, and slab selective excitation and flow suppression^36^. Bias-field corrected quantitative T1 maps and T1 images were obtained from images at the two inversion times (TI1/TI2).

### High resolution intracortical myelin profiling using T_1_ maps

Image preprocessing followed the procedures developed and validated previously^39,40^. Skull-stripping, background-marking and alignment (using rigid 6 degrees-of-freedom registration, normalized mutual information) to a 0.4 mm MNI template using the CBS Tools (https://www.nitrc.org/projects/cbs-tools) were applied on the T1 maps, T1-weighted and T2 images. No data were discarded due to poor quality data. Then, images were corrected for field inhomogeneities and matched to a template image^39^ using 3D Slicer (https://www.slicer.org). Images were filtered with arteries and dura segmentation, corrected for partial volume effects and segmented into gray matter, white matter and cerebrospinal fluid (CSF) using the Topology-preserving, Anatomy-Driven Segmentation (TOADS)^69^ and Multi-object Geometric Deformable Model (MGDM)^70^ segmentation algorithms as integrated in the CBS Tools. The CRUISE algorithm^71^ was applied in order to extract the cortex. Such an algorithm is robust to white matter lesions^72^. Capitalizing on the CBS Tools’ Volumetric Layering module^73^, the cortex was then dissected into 10 cortical depth-levels using a volume-preserving approach. This level-set approach generates one 2-dimensional surface per layer containing T_1_ values at each surface location. Next, the 2-dimensional level-sets were transformed into 3-dimensional representations of T_1_ values using the CBS Tools’ Profile Sampling Module. This transformation represents each vertex T-values of each level-set surface as a column of identical T_1_ values perpendicular to the surface^39,40^. The final result of this pipeline is 10 volumes of cortical profiles, one for each depth-level, for each of 7 patients with AN and 11 healthy controls. The whole process was completed within each individual's native space.

The cortex was then parcellated in the native space using Freesurfer (https://surfer.nmr.mgh.harvard.edu/). This parcellation was based on the pre-processed T1-weighted images alone (independent of the cortex extraction described above) and was used to obtain 148 cortical regions-of-interest (ROIs) based on the Destrieux atlas, that is particularly suitable for this type of investigation by separating gyri from sulci^73,74^. In each participant and for each cortical ROI, we estimated the absolute T_1_ value as an inverse index of myelin concentration at each depth-level: so, we obtained a matrix consisting of 148 total rows (ROIs) and 10 columns (i.e. each intracortical myelin depth-level) per participant.

### Statistical analyses

All demographic variables were individually checked for missing data or outliers. Normality and equality of variances were verified using Shapiro–Wilk and Levene’s tests. Group comparisons for age and BMI were carried out using two-sided Independent-samples t-tests.

With the aim of getting a single index of total intra-cortical myelin concentration per participant, we averaged the values of the 10 myelin depth-measures across all the ROIs. The mean total intra-cortical myelin concentration was compared between patients and controls using an analysis of covariance (ANCOVA) with the group as main factor and age as covariate. Further, a bootstrap post-hoc comparison based on 1000 replicates was carried out in order to get a more stable group comparison result.

A Principal Component Analysis (PCA) with an oblique rotation (Promax algorithm) was applied on the original T_1_ values matrix across all participants. This matrix contained of 2664 rows (148 myelin concentration values per participant, one value per ROI) and 10 columns (10 depth-levels). PCA was applied in order to reduce the number of computations and to deal with correlated predictors (i.e. T_1_ values between multiple cortical depth-levels). Bartlett’s Test of Sphericity has been used to check PCA assumptions. The number of components was established through a parallel analysis^75^ that compared the real eigenvalues obtained from the matrix to the eigenvalues obtained from a Monte-Carlo simulated matrix based on random data with the same size^76^. As output of parallel analysis, a scree plot of eigenvalues was used to set up a threshold of significance and reliability of components using the Kaiser criterion (i.e. eigenvalues > 1). Based on the number of components extracted, we averaged the T_1_ values in different analyses. Following PCA, independent comparisons between groups were carried out by means of ANCOVA with averaged T_1_ values as dependent variable, the group as the main factor, age as a covariate and a bootstrap post-hoc comparison based on 1000 replicates.

Finally, for exploratory analyses, we investigated relationships between T_1_ values, BMI, osmolality and duration of illness by means of non-parametric correlations (Spearman’s rho rank coefficient) with significant level set at p < 0.05 uncorrected.

All statistical analyses were carried out using JASP (https://jasp-stats.org/, v. 0.11).

### Intracortical myelin clusters by principal component analysis

Bartlett’s Test of Sphericity resulted significant (X^2^ (26) = 19,657.73, p < 0.001), suggesting a redundancy in the T_1_ values’ matrix. Two components (RC1 and RC2) were detected by parallel analysis using an eigenvalue threshold = 1 (Kaiser criterion) (see scree plot, Fig. 1SM). Table 4 shows the contribution of each depth-measure in each component.

**Table 4:** PCA results. Loading coefficients for RC1 and RC2 components.

Variables	RC 1	RC 2	Uniqueness
Layer 1		0.727	0.601
Layer 2		1.042	0.054
Layer 3		0.943	0.036
Layer 4		0.804	0.035
Layer 5	0.467	0.658	0.042
Layer 6	0.627	0.500	0.043
Layer 7	0.763		0.039
Layer 8	0.903		0.033
Layer 9	1.026		0.056
Layer 10	1.038		0.145

The first column reports all the variables included (T_1_ values in cortical depth-levels from 1 to 10). RC1 and RC2 columns represent variable loadings on that specific component. Uniqueness shows the percentage of the variance of each variable not explained by the component.

Specifically, T_1_ values at depth levels 1 to 5 contributed to RC2, whereas those in depth levels 6–10 contributed to RC1 (a path diagram is available in Fig. 1SM). In case of participation in both components (as in the case of measures in depth levels 5 and 6), the layer was associated with the component whose expression was greater. The path diagram and scree plot available in Fig. 1SM visually confirm the contribution of each depth measure to RC1 and RC2 and the reliability of the number of components extracted from the PCA.

## Supplementary Information

Supplementary Figure S1.

## Data Availability

The datasets generated during and/or analyzed during the current study are available from the corresponding author on reasonable request.
