# Supplementary material for: Initial evidence of abnormal brain plasticity in anorexia nervosa: an ultra-high field study
Source: Sci Rep. 2022 Feb 16;12:2589. doi: 10.1038/s41598-022-06113-x (PMC8850617; doi:10.1038/s41598-022-06113-x)

**Supplementary Information File**

**Initial evidence of abnormal brain plasticity in Anorexia Nervosa: an ultra-high field study**

Edoardo Pappaianni^1^, Bianca Borsarini^1^, Gaelle E. Doucet^2^, Ayelet Hochman^3^, Sophia Frangou^4,5^ & Nadia Micali^1,6,7*^

*Affiliations*

1. *Department of Psychiatry, Faculty of Medicine, University of Geneva, Geneva, Switzerland*
2. *Boys Town National Research Hospital, Omaha, Nebraska, USA*
3. *Department of Psychology, St. John’s University, Queens, New York, USA*
4. *Department of Psychiatry, Icahn School of Medicine at Mount Sinai, New York, NY, USA*
5. *Department of Psychiatry, University of British Columbia, Vancouver, BC, Canada*
6. *University College London, Great Ormond Street Institute of Child Health, London, UK*
7. *Department of Pediatrics, Gynecology and Obstetrics, Faculty of Medicine, University of Geneva, Geneva, Switzerland*

** Corresponding author:*

*Pr Nadia Micali*

*2 rue Verte, 1205 Genève*

*+41 223728955*

*nadia.micali@hcuge.ch*

*Figure 1SM. PCA results. a) Parallel analysis results. Scree-plot showing the reliability of the two components based on the eigenvalues threshold = 1 (Kaiser criterion). B) Path diagram showing the contribution of each depth-measure in each component (RC1-RC2).*


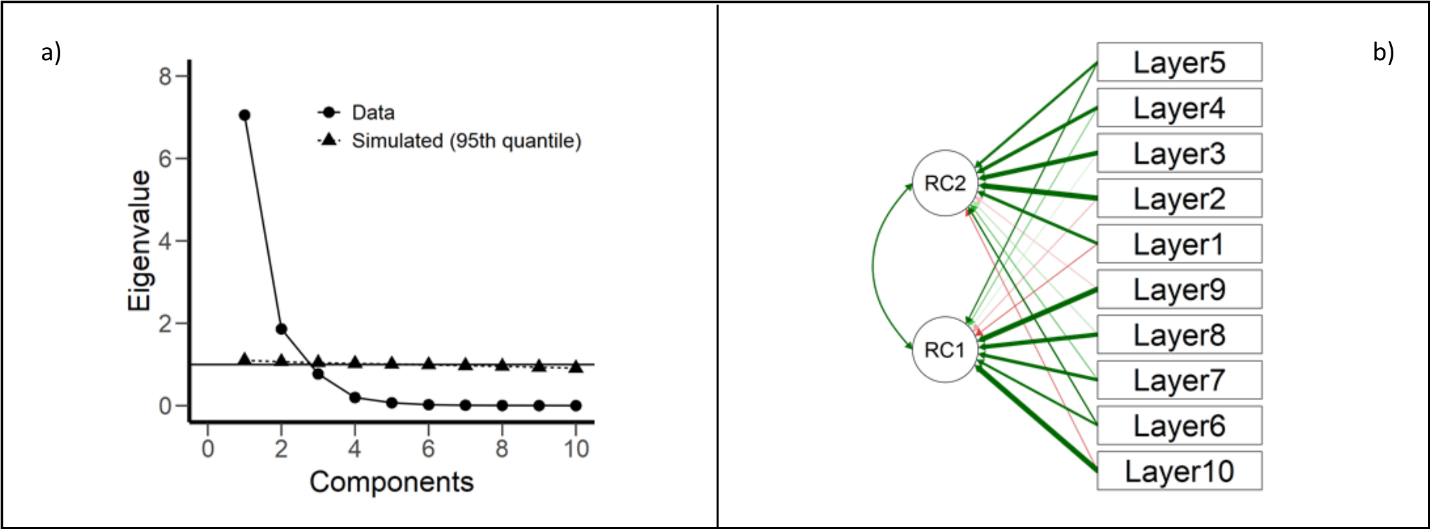

Supplement: Supplementary file 1 — Supplementary Figure S1. [file 41598_2022_6113_MOESM1_ESM.docx]
